# How can large-celled diatoms rapidly modulate sinking rates episodically?

**DOI:** 10.1093/jxb/eraa129

**Published:** 2020-03-12

**Authors:** Michel Lavoie, John A Raven

**Affiliations:** 1 Québec-Océan and Unité Mixte Internationale Takuvik Ulaval-CNRS, Département de Biologie, Université Laval, Québec-Océan, Québec, Canada; 2 Division of Plant Science, University of Dundee, The James Hutton Institute, Invergowrie, Dundee, UK; 3 School of Biological Sciences, University of Western Australia, Crawley (Perth), WA, Australia; 4 Climate Changer Cluster, University of Technology Sydney, Ultimo, NSW, Australia; 5 University of Essex, UK

**Keywords:** Cell volume, density, diatom, energy cost, episodic sinking, solute concentration

**Large variations in sinking rate of large-celled diatoms with a periodicity of ~10 s have their basis in cell density changes. Of the three mechanisms we model, the predicted energy cost is lower for periodic variations in the increase in cell volume than for variations in either the intracellular concentration of inorganic ions, or of organic solutes, that form solutions of different densities These predictions require experimental testing.**

Diatoms are silicified microalgae; marine planktonic diatoms account for at least 20% of global primary productivity, and 40% of the ‘biological pump’ that sequesters atmospheric CO_2_ as organic C in the deep ocean (Tréguer *et al.*, 2018). Small marine diatoms generally have a greater density than does seawater, and so they sink (Miklasz and Denny, 2010; Villareal *et al.*, 2014; Kemp and Villareal, 2018). The high density of the silicified frustules often cannot be offset by a low-density solution in the vacuole, due to constraints on: (i) the lowest possible density of the vacuolar solution, even with active water transport; and (ii) the fraction of the cell volume occupied by the vacuole (Boyd and Gradmann, 2002; Miklasz and Denny, 2010; Raven and Doblin, 2014; Lavoie *et al.* 2015). With increasing cell volume, and an increasing fraction of the cell volume occupied by the vacuole, the overall cell density can be lower than that of seawater, allowing positive buoyancy in some diatoms (Allen, 1932; Gross and Zeuthen, 1948; Villareal, 1988; Boyd and Gradmann, 2002; Raven and Doblin, 2014; Villareal *et al.*, 2014; Kemp and Villareal, 2018). This upward movement is particularly important for large-celled diatoms (e.g. *Ethmodiscus* spp. and *Rhizosolenia* spp.), which undergo periodic vertical migration in the oligotrophic ocean allowing them to exploit vertically (light at the surface and nutrients at depth) and temporally (photoperiod and scotoperiod) segregated resources in the water column (Raven and Doblin, 2014; Lavoie *et al.*, 2015). Increased density results from polysaccharide synthesis and the accumulation of ions generating dense solutions (e.g. K^+^), and decreased density results from the accumulation of ions generating less dense solutions (e.g. Na^+^) and by active water influx (e.g. Moore and Villareal, 1996; Raven and Doblin, 2014; Lavoie *et al*., 2015, 2016).

Recent ground-breaking studies of Gemmell *et al.* (2016) and Du Clos *et al.* (2019) showed that large-celled diatoms can control their sinking rate over time scales of seconds. These rapid oscillatory changes in the sinking rate occurred in three large-celled marine diatoms (*Coscinodiscus radiatus*, *Coscinodiscus wailesii*, and *Palmerina hardmaniana* with cell radii of 56, 123, and 112 μm, respectively). In illuminated N-depleted cultures of these algae, the sinking rate varied from 0.025 mm s^−1^ to 0.2 mm s^−1^ over a period of 5–10 s (Gemmell *et al.*, 2016). In the dark, these oscillations in sinking occurred at a lower frequency (Du Clos *et al.*, 2019). Such rapid high-frequency variations in sinking rate occurring via an as yet unknown mechanism could help alleviate diffusive limitation of nutrient uptake in oligotrophic marine environments (Du Clos *et al.*, 2019).

This fast periodic oscillatory behaviour in diatoms contrasts with other cyclical phenomena under constant conditions in *Coscinodiscus*, namely fluctuations in photosynthetic rate (Kühn and Raven, 2008) and in cell elongation (Olson *et al.*, 1986), which have lower frequencies than the variations in sinking rate in the light (Gemmell *et al.*, 2016). Hence, those phenomena alone cannot be related to the fast cyclical modulation of the diatom sinking rate. Here we discuss the feasibility and potential quantitative importance of three mechanisms potentially explaining the intriguing fast episodic changes in sinking of large-celled diatoms taking *Coscinodiscus wailesii* as a model species (Supplementary Protocol S1–S3 at *JXB* online). These mechanisms are (i) high-frequency modulation of Na^+^ and K^+^ permeability, as occurs in action potentials with transmembrane ion exchange; (ii) metabolism interconverting low-density organic cations and higher density cations; and (iii) fast cyclical changes in the cell expansion rate.

## Effects of three putative mechanisms driving fast oscillatory changes in the sinking rate of large-celled diatoms

The first mechanism involving a putative rapid Na^+^/K^+^ exchange at the plasmalemma using Na^+^ and K^+^ channels as used in action potentials and Na^+^-K^+^ ATPase in large-celled diatoms is explained and discussed in Box 1 and Supplementary Protocol S1. Our analysis suggests that this could only explain fast (~0.1 Hz) cyclical changes in the sinking rate of *C. wailesii* at a large energy cost.

Box 1. **Summary and energy cost of fast Na^+^/K^+^ exchange at the plasmalemma**

This strategy would first involve Na^+^ influx into the cytosol down the electrochemical gradient, particularly for diatoms with low cellular Na^+^ and high K^+^ such as *Coscinodiscus granii* (Kesseler, 1974) and *C. wailesii* (Melkikh and Bessarab, 2010), and depolarization of the inside-negative membrane electrical potential difference (Boyd and Gradmann, 1999). This would provide a driving force for K^+^ efflux through K^+^ channels, replacing higher density hydrated K^+^ ions (ρ=1034.3 kg m^−3^) with lower density hydrated Na^+^ ions (ρ=1028.4 kg m^−3^) in the cytosol (Boyd and Gradmann, 2002), and hence potentially decreasing cell sinking rate at no ATP running cost. In contrast, re-establishment of the initial low Na^+^ and high K^+^ concentration, which would increase the sinking rate, would need cellular energy, probably via the use of an electrogenic plasmalemma Na^+^-K^+^ ATPase, which apparently occurs in diatoms (Bhattacharya and Volcani, 1980; Rees, 1984; Flynn *et al.*, 1987) with energetically downhill K^+^ influx for charge balance. We performed mechanistic energy cost and vertical gravitational sinking calculations in Supplementary Protocol S1 for the large-celled diatom *C. wailesii* assuming a low cellular osmolarity similar to that of seawater, which maximizes the sensitivity of the sinking rate to a change in inorganic ion composition and decreases the energy cost per unit of sinking rate change. Those calculations indicate that an 8.8-fold change in sinking rate is coupled to large energy expenditure (48% added to the total energy cost of growth) due to an electrogenic 3Na^+^efflux, 2K^+^influx ATPase with energetically downhill 1K^+^ influx through a K^+^ channel, giving electroneutrality at the plasmalemma, and a similar mechanism at the tonoplast.

A second possibility involves the exchange of organic solutes yielding solutions of different densities. Based on the large energy cost of synthesizing such organic solutes in quantities modulating cell density (Supplementary Protocol S2), we also argue that a prohibitive energy cost occurs for rapid cyclical (~0.1 Hz) synthesis and breakdown of organic cations such as tetramethylammonium that yield a solution of low density; furthermore, tetramethylammonium and similar solutes have not been reported in diatoms.

Similar to the first strategy above, rhythmic changes in the rate of cell expansion through modulation of active water transport appear to be a plausible mechanism although at a significant energy cost (an additional 16% of the cost of growth) explaining fast cyclical (~0.1 Hz) changes in the sinking rate of large-celled diatoms such as *C. wailesii* (Box 2; Supplementary Protocol S3).

Box 2. **Summary and energy cost of episodic cell volume increase and active water transport**

Episodic increases in cell volume (Olson *et al.*, 1986; cf. Kühn and Raven, 2008) could modulate cell density and account for episodic sinking if cell volume increase is not driven by turgor requiring a continuous high cell osmolarity, but rather by cytoskeletal motors (Harold *et al.*, 1996; Pickett-Heaps and Klein, 1998; Raven and Doblin, 2014). Starting with a turgid cell with a greater density (density for the low and high osmolarity cases 1024.92 kg m^−3^ and 1039.52 kg m^−3^, respectively) than that of seawater (seawater density=1024.91 kg m^−3^), an increase in cell volume at a rate faster than ions are accumulated but not faster than water can enter [down the (decreased) water potential gradient resulting from the smaller difference in osmolarity between cell contents and the seawater medium] decreases the density of the cell. This decrease in cell osmolarity can continue until reaching seawater osmolarity, when a further increase in volume enclosed by the cell wall would lead to plasmolysis unless active water influx occurs. After the cessation of the cell expansion phase, continuing ion influx restores intracellular osmolarity and hence the cell density to maximum values, and the sinking rate increases too until the next cycle of cell expansion begins.

Mechanistic energy cost calculations coupled to modelling of the effect of changes in cell density on sinking rate (see Supplementary Protocol S3) suggest that the above strategy could occur at a significant, but potentially manageable, energy cost. Assuming a low cell osmolarity, which tends to minimize the cell energy cost for a given change in cell sinking rate, we found that modulating active water transport can modulate the sinking rate by >20-fold in *C. wailesii* at a cost related to water uptake equivalent to at least 16% of the total energy cost for growth. Even though the above differential cell expansion hypothesis strongly modulates the cell sinking rate in the range of 1×10^−4^ mm to 5×10^−6^ mm s^−1^, which is very much smaller than the range of absolute sinking rates measured by Gemmell *et al.* (2016) (between ~0.025 mm s^−1^ and 0.2 mm s^−1^), considering the difference in experimental conditions between Olson *et al.* (1986) and Gemmell *et al.* (2016), and the assumptions made in our model calculations (e.g. parameterization of structural components relying on empirical equations, inclusion of the putative main osmolyte glycine betaine), our novel hypothesis enabling fast vertical diatom displacement in the water column helps move forward our understanding of the factors explaining fast cyclical sinking in diatoms.

It must be emphasized that the observed fluctuations (but also frequency of measurements) in the rate of volume increase in large-celled diatoms are at a much lower frequency than is needed to explain the episodic sinking phenomenon through our active water transport hypothetical strategy (Box 2). Further experiments and modelling studies of unsteady sinking would be needed to test the presence of such a mechanism.

## Conclusion

The first mechanism discussed above, namely exchanging ‘heavy’ intracellular ions for ‘light’ extracellular ions to decrease the sinking rate, and vice versa for re-establishing the original faster sinking rate, can cost 48% of the energy cost of growth (Table 1). The organic solute exchange mechanism (strategy 2) would be even more costly (Table 1). The third mechanism, namely episodic cell volume increase with active water transport in parallel with steady solute uptake from seawater, can cost 16% of the total energy cost of growth (Table 1). According to our analysis, episodic increases in cell volume at the frequency of the changes in the rate of sinking has the smallest energy cost of the three mechanisms and thus appears most advantageous. Moreover, this strategy involving cytoskeletal motors for fast cyclical cell expansion is in line with the results of Gemmell *et al.* (2016), showing that episodic sinking of *C. wailesii* was eliminated by the myosin ATPase inhibitor 2,3-butane dione monoxine and by the actin inhibitor latrunculin A, applied separately, and that episodic sinking was restored after rinsing in filtered seawater. These findings are consistent with an essential role for the actomyosin mechanochemical motor in episodic sinking (Supplementary Protocol S3).

**Table 1.** Additional energy costs of episodic sinking relative to steady sinking for the three mechanisms

Mechanism	Additional energy requirement relative to growth with steady rate of sinking=100%
Downhill Na^+^ influx and K^+^ efflux, followed by energized Na^+^ efflux and K^+^ influx	~48%
Metabolism interconverting organic cations forming low-density solutions and organic cations forming higher density solutions	≥50%
Fast cyclical modulation of the rate of cell expansion and water uptake, with active water influx	≥16%

See text and Supplementary Protocols S1–S3 for details.

The changes in sinking rate in the light, as a function of nutrient availability, despite their energy cost, probably relate to balancing the supply of photons and nutrients that can be spatially separated, as suggested by Gemmell *et al.* (2016) and Du Clos *et al.* (2019). However, we have no further explanation other than those provided by Du Clos *et al.* (2019) for the presumably energy-costly very slow sinking with limited periodic variation in the dark under nutrient depletion. Even though it remains unknown whether or not one or several co-occurring mechanisms explain episodic sinking in diatoms, our analysis provides a new testable hypothesis useful for future laboratory experiments. No doubt the recent discovery of frequent variations in the sinking speed fluctuations in sinking of a planktonic diatom by Gemmell *et al.* (2016) will catalyse further research on large celled diatom physiology.

## Supplementary data

Supplementary data are available at *JXB* online.

Protocol S1. Downhill Na^+^ influx and K^+^ efflux followed by energized restoration of the initial ion content.

Protocol S2. Metabolism interconverting organic cations forming low-density solutions and organic cations forming higher density solutions.

Protocol S3. Fast cyclical modulation of the rate of cell expansion and water uptake, with active water influx.

eraa129_suppl_Supplementary_Data
