# Supplementary material for: How can large-celled diatoms rapidly modulate sinking rates episodically?
Source: J Exp Bot. 2020 Mar 12;71(12):3386–9. doi: 10.1093/jxb/eraa129 (PMC7364400; doi:10.1093/jxb/eraa129)
Supplement: eraa129_suppl_Supplementary_Data [file eraa129_suppl_supplementary_data.pdf]

Supplementary Data

S1: Downhill Na^+ influx and K^+ efflux followed by energized restoration of the initial ion content

Expanding on the summary in Box 1, there are a number of reports on the characteristics of plasmalemma ion channels in diatoms (Gradmann and Boyd, 1999, 2000; Taylor, 2009; Hedrich, 2019; Helliwell *et al.* 2019). The work on centric diatoms shows the occurrence of plasmalemma K^+ channels (Gradmann and Boyd, 1999, 2000) and Na^+ channels (Taylor, 2009; Hedrich, 2019; Helliwell *et al.* 2019). Activation of a Na^+ channel would permit Na^+ influx into the cytosol down the electrochemical gradient, particularly for diatoms with low cellular Na^+ and high K^+ such as *Coscinodiscus granii* (Kesseler, 1974) and *C. wailesii* (Melkikh and Bessarab, 2010), and depolarisation of the inside-negative membrane electrical potential difference (Boyd and Gradmann, 1999). This would provide a driving force for K^+ efflux through K^+ channels, replacing higher-density hydrated K^+ ions (ρ = 1034.3 kg m^{-3}) with lower-density hydrated Na^+ ions (ρ = 1028.4 kg m^{-3}) in the cytosol (Boyd and Gradmann, 2002), and hence potentially decrease cell sinking rate at no ATP running cost. Since the capacitance of biological membranes is about 10 mF per square meter or 0.01 Coulomb per volt per square metre (Raven, 1984), where 1 Coulomb is the positive charge of 10 μmole Na^+, and the *Coscinodiscus wailesii* plasmalemma has a usual inside-negative electrical potential differences of -0.06 - -0.09 V, with occasional excursions to as low as -0.165 V (Boyd and Gradmann, 1999), it follows that only around 10 nmole per square metre Na^+ influx would be needed for complete membrane depolarisation for a given action potential. This means that only 160 mol m^{-3} Na^+ per action potential would be taken up, given a *C. wailesii* cell surface area of 7.8 x 10^{-8} m^2 and cell volume of 4.9 x 10^{-12} m^3 (Melkikh

and Bessarab, 2010). Therefore multiple repetitive fast action potentials would be needed in order to significantly increase and decrease Na^{+} and K^{+} cellular concentrations (in the mol m^{-3} range), respectively. However, decreasing overall cellular K^{+} concentration would require maintenance of a high K^{+} in the cytosol, nucleoplasm, chloroplast and mitochondria for enzyme activity perhaps via transport of vacuolar K^{+} transport out of the cell (Raven, 2018). By contrast, re-establishment of the initial low Na^{+} and high K^{+} concentration, and hence the original sinking rate, would need cellular energy, probably via the use of a plasmalemma Na^{+} K^{+} ATPase, which apparently occurs in diatoms (Bhattacharya and Volcani, 1980; Flynn *et al.*, 1987; Rees, 1984). The use of a Na^{+}-H^{+} antiporter in parallel with a H^{+} efflux ATPase is less likely, in view of the small H^{+} electrochemical potential difference across the plasmalemma of *Coscinodiscus* (Melkikh and Bessarab, 2010).

What would be the quantitative effect of Na^{+}/K^{+} exchange on fast cyclical changes in sinking rate of large celled diatoms? Would it be energetically costly? Using the model of Lavoie *et al.* (2015) adapted for *C. wailesii*, here we computed the decrease in sinking rate due to Na^{+} influx and K^{+} efflux coupled to action potential-like downhill fluxes through ion channels, and estimate the energy cost related to the use of a Na^{+} K^{+} ATPase to restore the initial Na^{+} and K^{+} cellular composition. We used the modeling methodology described in Lavoie *et al.* (2015, 2016) with further details below in order to predict *C. wailesii* sinking rate or cell density as a function of cell biochemical composition and morphology. We find that the investment in cellular energy require for this strategy is probably high in order to sustain fast oscillations in sinking rates.

We ran our model assuming a high cellular osmolarity, i.e.18% higher than that of seawater (1195 osmole m^{-3}) as assumed in previous work for large celled diatoms (Boyd and Gradmann, 2002) and a low osmolarity (859.4 osmole m^{-3}, i.e., 15 % lower than that of seawater) to cover a range of possible osmolarities that should minimize or maximize, respectively, the sensitivity of sinking rate to changes in the cell osmolarity while keeping the cell negatively buoyant (i.e., cell density > seawater density). Note that for the low osmolarity case, we assume active water influx (Raven and Doblin 2014) to prevent plasmolysis. Osmolarity was adjusted with addition of the putative organic osmolyte glycine betaine (GBT) and by modulation of intracellular ions concentrations. The concentration of GBT was 105 mole m^{-3} or 0 mole m^{-3} for the high and low osmolarity case, respectively. The concentrations of structural components (proteins, lipids, carbohydrates) were first approximate with the empirical equations of Hitchcock (1982) and then adjusted so that the total sum of cellular organic carbon, including the osmolyte GBT and carbon from protein, lipid and carbohydrate equal the cellular C concentration modeled with the empirical equation of Strathmann (1967) (2.82×10^{-4} pg C / cell) (mass balance within 1%). We consider that the mass of carbon accounts for 52, 40 and 85% of the protein, carbohydrate and lipid mass, respectively (Boyd and Gradmann, 2002).

For both osmolarity cases, to compute total cell density, we consider a model *C. wailesii* cell radius of 1.25×10^{-4} m, a cell height of 1×10^{-4} m (volume = $4.91.10^{-12}$ m^3; area = $7.86.10^{-8}$ m^2) (Melkikh and Bessarab, 2010). For the high osmolarity case, we used the inorganic cellular ion composition measured in *C. wailesii* by Kesseler (1974), i.e., cellular Na^+, Cl^-, K^+, Ca^{2+}, Mg^{2+}, SO_4^{2-} concentrations of 125, 450, 461, 19, 19, 118 mol m^{-3}, respectively (charge balance agrees within 3%). However, for

the low osmolarity case, we assume a combinations of ions that yield a cell density (1024.916 kg m^{-3}) quasi-identical to that of seawater (1024.91 kg m^{-3}), i.e., cellular Na^{+}, Cl^{-}, K^{+}, Ca^{2+}, Mg^{2+}, SO_4^{2-} concentrations of 125, 450, 387.4, 0, 0, 0 mole m^{-3}, respectively (charge balance agrees within 12%). For both osmolarity cases, we assume a negligible NO_3^{-} and NH_4^{+} concentration as measured by Kesseler (1974) since the cells are N depleted.

The model cell sap density of 1036.90 and 1022.2 kg m^{-3} for the high and low osmolarity, respectively, is close to (± 0.8%) or within the range of measured cell sap density in *C. wailesii*, i.e., between 1015.0 and 1028.7 kg m^{-3} (Villareal, 1988). The total cell density was 1039.52 and 1024.92 kg m^{-3} for the high and low osmolarity case, respectively. Under those conditions, the rate of sinking of the cells is 0.33 mm s^{-1} (high osmolarity) and 1.3 x 10^{-4} mm s^{-1} (low osmolarity).

For the high osmolarity case, the model predicts that a 100 mol m^{-3} increase in Na^{+} coupled to a 100 mol m^{-3} decrease in K^{+} only decreased the sinking rate of *C. wailesii* by 4% while a 200 mol m^{-3} or 400 mol m^{-3} Na^{+} / K^{+} swap only decreased cell sinking rate by 8% or 19%, respectively. Such a very high ion exchange on a time scale of around 10 seconds translate into a Na^{+} uptake flux of up to 2.5 mol m^{2} s^{-1}, which would approach diffusive limit of Na^{+} uptake by the cell (around 6 mol m^{-2} s^{-1} for an equivalent cell radius of 105 μm and a Na^{+} diffusion coefficient of 1.33 x 10^{-9} m^{-2} s^{-1} measured by Li and Gregory (1974)). The cellular Na^{+} concentration would also approach the seawater Na^{+} concentration, which would decrease the concentration component of the driving force for uptake. For the low osmolarity case, a much smaller swap of only 0.9 mol m^{-3} Na+ for 0.9 mol m^{-3} K^{+} decreased sinking rate by as

much as 8.8-fold, which is a trend consistent with the results of Gemmell *et al.* (2016).

Is restoring the initial low Na^+ and high K^+ through the use of a Na^+ K^+ ATPase costly in terms of energy? Taking a total cellular C of 2.8 x 10^{-8} mol C $cell^{-1}$ calculated above and the need for 24 mol absorbed photons per mol particulate organic C as measured in *Chlamydomonas reinhardtii* by Kliphuis *et al.* (2012) and predicted by mechanistic modeling of algal bioenergetics with some allowance for lack of waste minimization (Raven *et al.*, 2014; Raven and Ralph, 2015), we calculate that 6.72 x 10^{-7} mol photons are needed per cell doubling. For the low osmolarity case, a Na^+ release flux of 0.9 mol m^{-3} to re-establish baseline low Na^+ cellular concentration using an electrogenic Na^+ K^+ ATPase (1 mole ATP per 3 mole Na^+ release and 2 mole K^+ taken up, with 1 mole K^+ entering energetically downhill by K^+ channels giving electroneutrality) would cost 5.03 x 10^{-12} mol ATP per cell per around 10 seconds. Taking into account that the cell will oscillate between period of Na^+ uptake / K^+ efflux and period of Na^+ efflux / K^+ uptake of 10 seconds each, it follows that 4.35 x 10^{-8} mole ATP per cell will be required per 24 h. Assuming 10 mole photon (400-700 nm) per mol CO_2 converted to 1 mol carbohydrate, including the energy costs of the CO_2 concentrating mechanism (Raven *et al.* 2014), and 2.7 mol ATP produced in complete oxidation of 1 mole carbohydrate (Raven and Beardall, 2017), 3.7 mole photons (10/2.7 = 3.7) are needed to produce 1 mole ATP. 4.35 x 10^{-8} mole ATP per cell per 24 h thus requires 1.6 x 10^{-7} mole absorbed photon per cell per 24 h. For a cell doubling of 24 h in *C. wailesii* (Olson *et al* 1986) or longer under N limitation, an increase of 24% (16 x 100 / 67.2) of the absorbed photon cost for growth would be

required just to fuel the Na^+ K^+ ATPase for the low osmolarity case. The additional energy cost is greater for the high osmolarity case.

Furthermore, energy is required for moving K^+ and Na^+ between cytosol and vacuole, where most of the cellular K^+ and Na^+ occurs. Less is known of the energetics of these fluxes than of those at the plasmalemma (Bussard and Lopez 2014; Raven and Doblin 2014; Schreiber et al. 2017), but they could be as high as those at the plasmalemma, giving an additional cost of 48% of the energy cost of organic matter production in cell growth. These calculations suggest that this strategy involving fast Na^+ / K^+ exchange at the plasmalemma and tonoplast for episodic sinking rate modulation in large celled diatoms is possible, but might not be energetically sustainable. Our calculations do not rule out however the use of Na^+ / K^+ action potentials be use as a signal triggering the mechanisms that rapidly modulate buoyancy changes.

S2: Metabolism interconverting organic cations forming low density solutions and organic cations forming higher density solutions

This possibility is based on a role for organic cations, and especially tetramethylammonium (= tetramine), yielding low density solutions (Boyd and Gradmann, 2002; Lavoie *et al.*, 2016; Raven and Doblin, 2014) and decreasing cell density, and conversion of this cation into a cation forming higher-density solutions, increase cell density; reconversion to tetramethylammonium once more decreases cell density. Tetramethylammonium has been reported from the terrestrial flowering plant *Courbonia virgata* (Henry 1948) and in some sea anemones and predatory marine gastropods (Mathews et al. 1960; Anthoni et al. 1989; Turner et al. 2018). In addition to the absence of information on the occurrence of tetramethylamonium (or

related compounds yielding low-density solutions) in diatoms, the pathway of catabolism of tetramethylammonium to, (ex hypothesis), a denser product from which tetramethylammonium can be resynthesized, is not clear (Urakami *et al.*, 1990). Even assuming tetramethylammonium occurs in algae, its synthesis in parallel with cell growth in the giant-celled centric diatom *Ethmodiscus rex* at a concentration required for positive buoyancy consumes over half as much energy (expressed as absorbed photons used in photosynthesis to synthesise the particulate organic matter of the cell) (Lavoie et al. 2016), i.e. a total energy cost of more than 1.5 times that of particulate organic matter synthesis. The variations in tetramethylammonium concentrations needed to cause the alterations in cell density and explain episodic sinking would presumably require an even greater energy input, especially considering the high frequency of changes in sinking rate. This would be especially the case if tetramethylammonium efflux to seawater occurs, with replacement by a denser inorganic anion, as a means of increasing density, with a subsequent synthesis of more tetramethylammonium and efflux of the dense inorganic cation, is the explanation of the density changes needed for episodic changes in sinking rate.

S3: Fast cyclical modulation of the rate of cell expansion and water uptake, with active water influx

An alternative to the previously discussed ion exchange mechanism would be to exploit periodic cell and protoplasm expansion at a rate faster than ion uptake in order to explain the enigmatic high frequency episodic variations in sinking of large celled diatoms. The cell volume of *Coscinodiscus* sp. indeed expands episodically due to an increase in the length of the centric diatom cells by decreased overlap of the frustules while the cell radius stay constant (Olson *et al.*, 1986). However, small episodic

changes in cell volume at high frequency (on the order of seconds) in order to explain the fast sinking rate modulation recorded by Gemmell *et al.* (2016) and Du Clos *et al.* (2019) have not been demonstrated. The occurrence of high frequency variations in the rate of increase of length of the protoplasm and frustule could modulate cell density and account for episodic sinking, provided the increase in cell volume is not driven by turgor, which depends on cell osmolarity in excess of that in the medium, but rather by cytoskeletal motors (Harold *et al.*, 1996; Pickett-Heaps and Klein, 1998; Raven and Doblin, 2014). Starting with a turgid cell with a greater density (density for the low and high osmolarity case is 1024.92 kg m^{-3} or 1039.52, respectively), than that of seawater (seawater density = 1024.91 kg m^{-3}), increase in cell volume at a rate faster than ions are accumulated but not faster than water can enter (down the (decreased) water potential gradient resulting from the smaller difference in osmolarity between cell contents and the seawater medium) decreases the density of the cell. This decrease in cell osmolarity can potentially continue until reaching seawater osmolarity, at this point, no further net water uptake can occur and hence dilution of intracellular ions is no longer possible. After the rapid cell expansion phase, cell expansion rate could become slower than ion uptake. In this case, as continuing ion influx gradually increases again the intracellular osmolarity and hence the cell density to maximum values, the sinking rate increases too until the next cycle of protoplast and/or cell expansion starts again.

For the energy cost of cell expansion, the involvement of the actomyosin system mentioned above is supported by the demonstration in Figure 4 of Gemmell et al. **(**2016) that episodic sinking of *Coscinodiscus wailesii* was eliminated by the myosin ATPase inhibitor 2,3 butanedione monoxine (BDM) and by the actin inhibitor latrunculin A, applied separately, and that the episodic sinking was restored after rinsing the cells in filtered seawater, consistent with involvement of the actomyosin

mechanochemical motor in the episodic sinking. If the actomyosin system is involved in decreasing the overlap of the frustules, and the mean rate of frustule overlap is the same with and without episodic extension means identical energy costs of episodic and non-episodic extension, although episodic elongation sinking may need more actomyosin than a constant rate of elongation and hence sinking. More plausibly, rapid but episodic elongation could require a more rapid specific reaction rate of myosin ATP hydrolysis and/or longer stroket length in the actomyosin system during the rapid cell elongation phase. However, the mean sinking rate is increased (reversibly) by the two actomyosin inhibitors relative to controls, so the inhibitors may act on other sinking-related processes, either by acting on actomyosin or in other ways. The occurrence of actomyosin-based cytoplasmic streaming in characaean algae is controlled by action potentials (Beilby and Casanova MT 2014); as indicated in the main text, action potentials are known in diatoms. Overall, the discussion in this paragraph suggests that there need not be additional energy input to account for episodic rather than continuous cell expansion.

Here we used the *C. wailesii* sinking rate model (see details in the first section of Supplementary Information) in order to explore the sensitivity of sinking rate to the aforementioned differential cell expansion hypothesis. For a cell doubling time of 24 h (Olson *et al* 1986), here we speculate that a cyclical cell expansion of 0.023 % occurs each 10 sec followed by a 10 sec. period of no growth. We computed the effect of the associated 0.023% decrease in cell osmolarity on sinking rate due to episodic cell expansion for a rapid small increase in cell length by 0.023%. For the high osmolarity case, we found that decreasing cell osmolarity by 0.023% coupled to an equivalent increase in cell length did not decrease the sinking rate significantly. However, for the low osmolarity case, this cell expansion effect could decrease by 25-fold the cell sinking rate. In this condition, the cell density is similar to the density of seawater. Since those two densities are subtracted in Stokes law, changes in cell sinking rate in response to each incremental change in cell osmolarity (and thus cell

density) becomes disproportionately greater when the cell density is similar to seawater density (Lavoie *et al.*, 2015). Therefore, it appears that a rapid increase in the volume enclosed by the frustules and correspondingly increase in protoplast volume, combined with a constant rate of K^+, Cl^- and Na^+ (and other ions) influx, would decrease the sinking rate. After the first 10 s. phase of cell expansion, ion uptake rate could continue at the same rate than in the first 10 seconds phase while the cell volume stays constant (no expansion in the second 10 s phase) and density and sinking rate increase.

The above differential cell expansion strategy must be performed at a significant energy cost, which is potentially low (or at least manageable by the cell) according to the following reasoning. Here, it is assumed that active water transport occurs at the tonoplast, as in Raven and Doblin (2014). Here passive (downhill) water movement from the seawater medium to the cytosol using aquaporins in the plasmalemma, is assumed, requiring a higher osmolarity in the cytosol (and the compartments therein) than in seawater and the vacuole. Active water transport equates the water potential in the vacuole and the cytosol, maintaining the ratio of the volume of the cytoplasm relative to that of the vacuole.

The assumed volume expansion of 0.023% in a 10s period corresponds to an absolute cell volume increase of $1.13.10^{-15}$ m^3 (0.023% multiplied by total cell volume of $4.91.10^{-12}$ m^3, equals $1.13.10^{-15}$ m^3). With 55.55 kmole water per m^3, the water flux is $6.27.10^{-11}$ mole water. The cation-chloride transporter moves 500 mole water per mole (K-Na-2Cl), and restoring the gradient of one mole (K-Na-2Cl) gradient requires 2 mole ATP (Raven and Doblin 2014) or 250 mole water per mole ATP. The influx of

$6.27.10^{-11}$ mole water requires $6.27.10^{-11}/250 = 2.5.10^{-13}$ mole ATP per cell. This calculation ignores the additional energy cost of compensating for any water backflux down the water free energy (water potential) gradient from the medium into the cytoplasm.

Assuming 2 mole absorbed photons in cyclic electron flow and proton pumping in diatoms (1 mole electron per mole photon; 2 mole protons per electron [no protons pumped in Ferredoxin to Plastoquinone electron transport]; 4 mole protons per mole ATP: Larkum et al. 2017), the active water transport over 10s costs 5.10^{-13} mole absorbed photons. Since active water transport only occurs (in our model) for half the time, the photon cost for a cell doubling is 5.10^{-13} x 12 x 3600 = $1.08.10^{-7}$ mole photon per cell. The photon cost of cell doubling with 24 mole photons per mole cell C (Kliphuis et al. 2012), and $2.8.10^{-8}$ mole C per cell (Strathmann et al. 2017), is $6.72.10^{-7}$ mole absorbed photo per cell. Therefore, the minimum computed cost of active water transport ($1.08.10^{-7}$ mole absorbed photon per cell) represents a16% increase of the total energy cost for growth of $6.72.10^{-7}$ mole absorbed photon per cell.

References

Anthoni L, Bohlin L, Larsen C, Nielsen P, Nielsen N H, Christophersen C. 1989. Tetramine: occurrence in marine organisms and pharmacology **27,** 707-716.

Beilby MJ, Casanova MT 2014 The Physiology of Characean Cells. Springer.

Bhattacharya P, Volcani BE. 1980. Sodium-dependent silicate transport in the apochlorotic marine diatom *Nitzschia alba* (Na^+ gradient/Na^+,K^+-ATPase/membrane vesicles). Proceedings of the National Academy of Science USA **77**, 6386-6390.

Boyd CM, Gradmann AED. 1999. Electrophysiology of the marine diatom *Coscinodiscus wailesii* I. Endogenous changes of membrane voltage and resistance. Journal of Experimental Botany **50**, 445-452.

Boyd CM, Gradmann AED. 2002. Impact of osmolytes on buoyancy of marine phytoplankton. Marine Biology **141**, 605-618.

Bussard A, Lopez PJ. 2014. Evolution of vacuolar pyrophosphatases and vacuolar H^+-ATPase in diatoms. Journal of Marine Science and Technology **22,** 50-59.

Du Clos KT, Karp-Boss L, Villareal TA, Gemmell BJ. 2019. *Coscinodiscus wailesii* mutes unsteady sinking in dark conditions. Biology Letters **15**, 20181086.

Flynn KJ, Öpik H, Syrett PJ. 1987. The isolation of plasma membrane from the diatom *Phaeodactylum tricornutum* using an aqueous two-polymer phase system. Journal of General Microbiology **133**, 93-101.

Gemmell BJ, Buskey EJ, Villareal TA. 2016. Dynamic sinking behaviour in marine phytoplankton: rapid changes in buoyancy may aid in nutrient uptake. Proceedings of the Royal Society Series B **283**, 20161126.

Gradmann D, Boyd CM. 1999. Electrophysiology of the marine diatom *Coscinodiscus wailesii*. II. Potassium currents. Journal of Experimental Botany **50**, 453-459.

Gradmann D, Boyd CM. 2000. Three types of membrane excitations in the marine diatom *Coscinodiscus wailesii*. Journal of Membrane Biology **175**, 149-160.

Harold RL, Money FP, Harold FM. 1996. Growth and morphogenesis in *Saprolegnia ferax*: is turgor required? Protoplasma **191**, 105-114.

Hedrich R. 2019. Diatom signaling: A novel channel type Identified. Current Biology **29**, R317–R339.

Helliwell KE, Chrachri A, Koester JA, Wharam S, Verret F, Taylor AR, Wheeler GL, Brownlee C. 2019. Alternative mechanisms for fast Na^+/Ca^{2+} signalling in eukaryotes via a novel class of single-omain voltage-gated channels. Current Biology **29**, 1503-1511.

Henry AJ. 1948. The toxic principles of *Courbonia virgata*: its isolation and identification as a tetramethylammonium salt. British Journal of Pharmaclogy and Chemotherapy **3,** 187-188.

Hitchcock GL. 1982. A comparative study of the size-dependant organic composition of marine diatoms and dinoflagellates. Journal of Plankton Research **4**, 363-377.

Kesseler H. 1974. Die anorganisch-chemische Zusammensetzung des Zellsafies von *Coscinodiscus granii* (Bacillariophyceae, Centrales). Helgoläinder wissenschaftlisches Meeresuntersuntersuchungen **26**, 481-489.

Kliphuis AMJ, de Winter L, Vejrazka C, Klok AJ, Martens DE, Lamers PP, Janssen M, Wijffels RH. 2012. Metabolic modelling of *Chlamydomonas reinhardtii*: energy requirements for photoautotrophic growth and maintenance. Journal of Applied Phycology **24**, 253-266.

Larkum AWD, Ritchie RJ, Raven JA. 2018. Living off the Sun: chlorophylls, bacteriochlorophylls and rhodopsins. Photosynthetica **56,** 11-43.

Lavoie M, Levasseur M, Babin M. 2015. Testing the potential ballast role for dimethylsulfoniopropionate in marine phytoplankton: a modeling study. Journal of Plankton Research **37**, 699-711.

Lavoie M, Raven JA, Levasseur M. 2016. Energy cost and putative benefits of cellular mechanisms modulating buoyancy in aflagellate marine phytoplankton. Journal of Phycology **52**, 239-251.

Li YH, Gregory S. 1974. Diffusion of ions in seawater and deep-sea sediments. Geochimica et Cosmochimica Acta **38**, 703-714.

Mathias A P, Ross D M, Schachter M. 1960. The distribution of 5-hydroxytryptamine, tetramethylammonium, homarine, and other substances in sea anemones. Journal of Physiology **151,** 296-311.

Melkikh AV, Bessarab DS. 2010. Model of active transport of ions through diatom cell biomembrane. Bulletin of Mathematical Biology **72**, 1912-1924.

Olson RJ, Watras C, Chisholm SW. 1986. Patterns in individual cell growth in marine centric diatoms. Journal of General Microbiology **132**, 1197-1204.

Pickett-Heaps JP, Klein AG. 1998. Tip growth in plant cells may be amoeboid and not generated by turgor pressure. Proceedings of the Royal Society Series B **265**, 1453-1459.

Raven JA. 1984. Energetics and Transport in Aquatic Plants. New York, NY, USA: A. R. Liss.

Raven JA. 2018. The potential effect of low cell osmolarity on cell function through decreased concentration of enzyme substrates. Journal of Experimental Botany **69** 4667–4673.

Raven JA, Beardall J. 2017. Consequences of the genotypic loss of mitochondrial Complex I in dinoflagellates and of phenotypic regulation of Complex I content in other photosynthetic organisms. Journal of Experimental Botany **68**, 2683–2692.

Raven JA, Beardall J, Giordano M. 2014. Energy costs of carbon dioxide concentrating mechanisms in aquatic organisms. Photosynthesis Research **121**, 111-124.

Raven JA, Doblin. 2014. Active water transport in unicellular algae: where, why, and how. Journal of Experimental Botany **65**, 6279-6292.

Raven JA, Ralph PJ. 2015. Enhanced biofuel production using optimality, pathway modification and waste minimization. Journal of Applied Phycology **27**, 1-31.

Rees TAV. 1984. Sodium dependent photosynthetic oxygen evolution in a marine diatom. Journal of Experimental Botany **35**, 332-337.

Schreiber V, Dorsch J, Puzik K, Bäcker O, Liu X, Stork S, Schulz J, Heimerl T, Klingl A, Zauner S, Maier UG. 2017. The central vacuole of the diatom *Phaeodactylum tricornutum*: identification of new vacuolar membrane proteins and of a functional Di-leucine-based targetting motif. Protist **168,** 271-282.

Strathmann R. 1967. Estimating organic carbon content of phytoplankton from cell volume or plasma volume. Limnology and Oceanography **12**, 411-418.

Taylor AR. 2009. A fast Na^+/Ca^{2+}-based action potential in a marine diatom. Plos One **E4**, e4966.

Turner A H, Craik D J, Kaas Q, Schroeder C I. 2018. Bioactive compounds isolated from predatory marine gastropods. Marine Drugs **16,** article 118.

Urakami T, Araki H, Kobayashi H. 1990. Isolation and identification of tetramethylammonium-biodegrading bacteria. Journal of Biotechnology and Bioengineering **70**, 41-44.

Villareal TA. 1988. Positive buoyancy in the oceanic diatom *Rhizosolenia debyana* H. Peragallo. Deep-Sea Research Part A-Oceanographic Research Papers **35**, 1037-1045.

o
